# *In situ* Probe Microphone Measurement for Testing the Direct Acoustical Cochlear Stimulator

**DOI:** 10.3389/fnins.2017.00450

**Published:** 2017-08-15

**Authors:** Christof Stieger, Yasser H. Alnufaily, Claudia Candreia, Marco D. Caversaccio, Andreas M. Arnold

**Affiliations:** ^1^ARTORG Center, Artificial Hearing Research, University of Bern Bern, Switzerland; ^2^University Department of ENT, Head and Neck Surgery, Inselspital, University Hospital of Bern Bern, Switzerland; ^3^Department of ENT, University Hospital Basel Basel, Switzerland

**Keywords:** DACS, Codacs, microphone, intra-operative test, laser doppler vibrometer

## Abstract

**Hypothesis:** Acoustical measurements can be used for functional control of a direct acoustic cochlear stimulator (DACS).

**Background:** The DACS is a recently released active hearing implant that works on the principle of a conventional piston prosthesis driven by the rod of an electromagnetic actuator. An inherent part of the DACS actuator is a thin titanium diaphragm that allows for movement of the stimulation rod while hermetically sealing the housing. In addition to mechanical stimulation, the actuator emits sound into the mastoid cavity because of the motion of the diaphragm.

**Methods:** We investigated the use of the sound emission of a DACS for intra-operative testing. We measured sound emission in the external auditory canal (P_EAC_) and velocity of the actuators stimulation rod (V_act_) in five implanted ears of whole-head specimens. We tested the influence various positions of the loudspeaker and a probe microphone on P_EAC_ and simulated implant malfunction in one example.

**Results:** Sound emission of the DACS with a signal-to-noise ratio >10 dB was observed between 0.5 and 5 kHz. Simulated implant misplacement or malfunction could be detected by the absence or shift in the characteristic resonance frequency of the actuator. P_EAC_ changed by <6 dB for variations of the microphone and loudspeaker position.

**Conclusion:** Our data support the feasibility of acoustical measurements for in situ testing of the DACS implant in the mastoid cavity as well as for post-operative monitoring of actuator function.

## Introduction

The direct acoustic cochlear stimulator (DACS, commercial name: “Codacs”) is an implantable hearing system that was designed for patients suffering from moderate to severe combined conductive hearing loss. In combined hearing loss the transmission via the ossicular chain (malleus, incus, stapes) and the inner ear (cochlea) are defective. The DACS is currently investigational. The DACS was first implanted in four patients with otosclerosis, a disease that hinders sound transmission in the middle ear (Hausler et al., 2008). In further clinical studies, the DACS proved to be among the most powerful acoustic implants with maximal possible power output beyond patients uncomfortable loudness levels (Zwartenkot et al., 2014). The DACS device works on the principle of a mechanically actuated piston prosthesis: The DACS's vibrating rod replaces the long process of the incus, where a piston prosthesis, inserted into a stapes fenestration, is connected.

**Figure 1:**
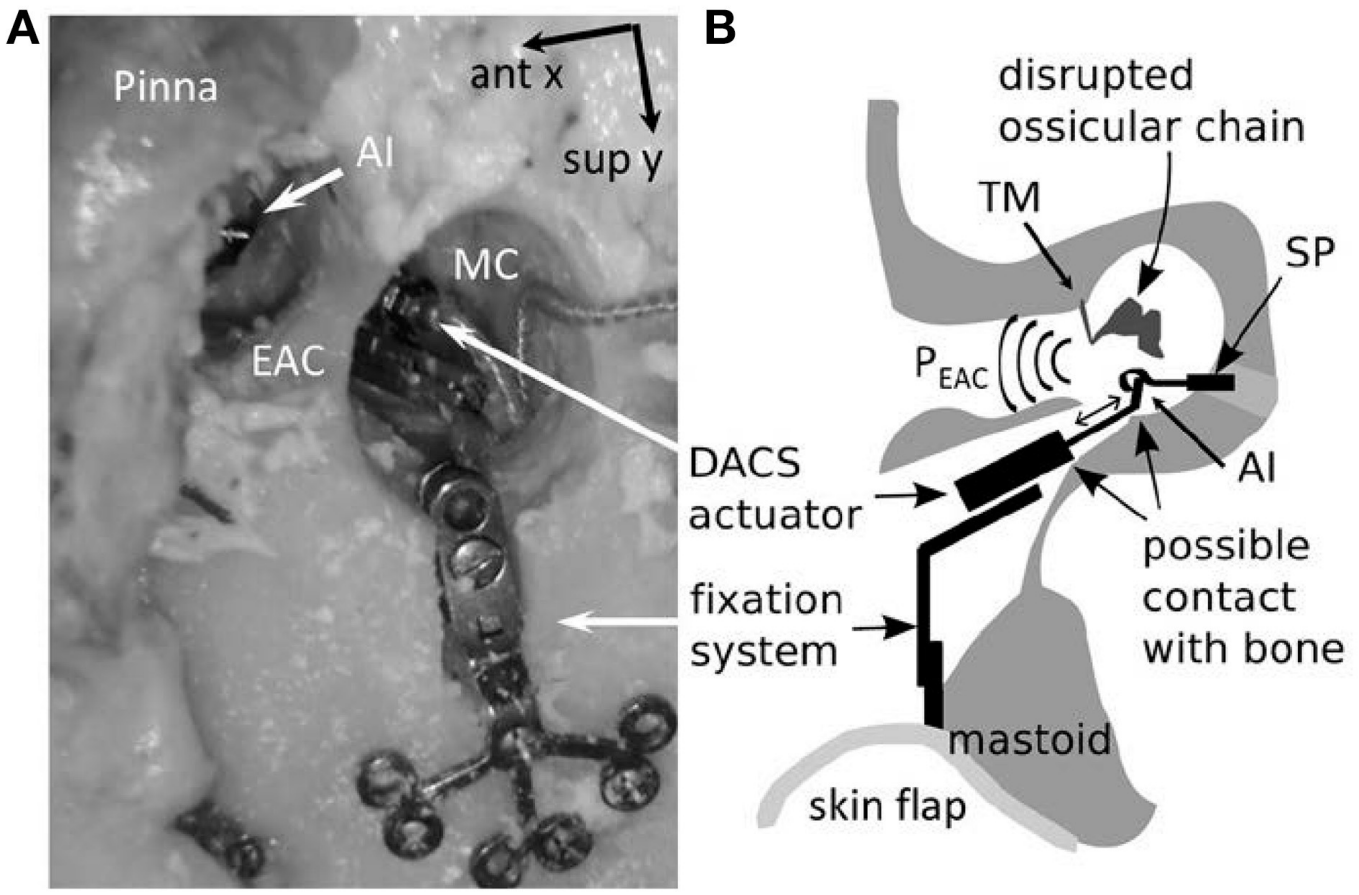
Intraoperative representation of the DACS (Direct acoustic cochlear stimulation) as photograph in a human cadaver **(A)** and in a schematic drawing **(B)**. The pinna is flapped and a cavity in the mastoid (MC) is performed behind the external auditory canal (EAC) in order to position the fixation system and the DACS actuator. The tympanic membrane (TM) is elevated and ossicular chain interrupted. A piston prosthesis (SP) crimped on the artificial incus (AI) of the DACS transmits the vibrations to the inner ear. The sound emission of the DACS P_EAC_ is propagated directly through the middle ear cavity to the EAC. Misplacement may happen most frequently in the narrow space of the facial recess leading to contact to the surrounding bone (indicated with arrows). ant x: anterior direction is defined as x-axis, sup y: superior direction is defined as y-axis.

The actuator of the DACS is held in the mastoid cavity with a dedicated fixation system (Figure 1).

During surgery, a piston prosthesis is crimped onto a lateral extension of the vibrating rod of the actuator (also called the artificial incus), which transmits the output of the implant to the cochlea. If the actuator is subject to significant mechanical stress during surgery, or if it is implanted with a portion of the rod touching the surrounding tissue, the output to the cochlea may be impaired. Therefore, an intra-operative test of proper actuator function is desirable to ensure free movement of the piston prosthesis.

In the pilot clinical study for DACS, laser Doppler vibrometry (LDV) was used to measure the rod vibration of the actuator (Bernhard et al., 2011). LDV measurements were also used to evaluate the equivalent sound-pressure level (ESPL) of the DACS actuator output as well as the influence of coupling on the actuator's motion (Chatzimichalis et al., 2012). LDV has become a standard tool in clinical and experimental middle ear research as well as mechanical implant evaluation (Rodriguez Jorge et al., 2006; Rosowski et al., 2007; Tringali et al., 2009; Arnold et al., 2010).

LDV has the advantage of being contactless and highly sensitive to the vibration of the target while being insensitive to ambient acoustical noise. On the other hand, there are a number of drawbacks of LDV in clinical environments. Due to high costs, not all implantation centers are equipped with an LDV device. The magnitude of the LDV signal is sensitive to the angle between the laser beam and the direction of motion of the measured object. The anatomy of the ear does not always permit setting the optimal measurement angle (Chien et al., 2006; Sim et al., 2010). The magnitude of the LDV signal also relies on the quality of the reflection, and liquid surrounding the implant may alter the reflection. Slight movements of the patient or vibrations of the microscope can result in a noisy LDV signal or cause the laser beam to be off target.

As an alternative, microphones can be used for testing middle ear implants. Sound pressure measurements in the external auditory canal P_EAC_ (referred to hereafter as the ear canal) or at the surface of the scalp have been used during middle ear transducer or floating mass transducer implantation surgeries (Winter et al., 2002; Jenkins et al., 2007). In both cases, the actuator is coupled to the ossicle chain. The main effect of these actuators is to drive the cochlea through motion of the stapes. A side effect is the generation of P_EAC_ due to the stimulation of the ossicle chain in the reverse direction. This sound can be used for monitoring the function of the implant (Winter et al., 2002).

The DACS stimulates the inner-ear fluid directly via a piston prosthesis attached to the vibrating rod and thus bypasses the middle ear structures. The implantation procedure includes a total or partial removal of the stapes (Hausler et al., 2008; Lenarz et al., 2013).

A unique element of the DACS actuator is its titanium diaphragm, which seals the casing of the electromagnetic components hermetically while enabling the movement of the stimulation rod. The diaphragm emits sound into the mastoid cavity, which is then transmitted to the ear canal. In short, the DACS actuator itself is a sound source that can be measured with an ear canal microphone. This measurement setup could permit direct testing of the actuator function after insertion into its fixation system. The surgeon could confirm that the delicate diaphragm is not damaged during insertion and that the vibrating rod is moving freely. The tympanic membrane is typically elevated during surgery (Figure 1B). As a consequence, the resulting P_EAC_ during the intra-operative scenario is solely due to the sound emitted by the diaphragm of the actuator. In a post-operative scenario, the tympanic membrane is closed, separating the ear canal from the middle ear cavity, and skin and subcutaneous tissue cover the surgically drilled mastoid cavity. The actuator acts as a sound source that stimulates the middle ear and the tympanic membrane in the reverse direction.

In this experimental study, we investigated microphone measurements to characterize the actuator's correct function and to use the findings as an instrument to test the integrity of the implant during surgery and for post-operative examination of the function of the actuator. As such, we examined the relationship between the velocity of the actuator vibrating rod (V_act_) measured by LDV and the sound emission measured by a microphone (P_EAC_). Additionally, we studied a series of parameters that may influence acoustical measurements, including one example of misplacement and a measurement of an actuator failure with a device which performed out of the defined specifications.

## Materials and methods

Thiel-embalmed cadaver heads were used in this study. The specimens were provided by the local department of Anatomy. The local ethics committee approved the use of the Thiel-embalmed heads for research (KEK-BE 030/08). Thiel-embalmed cadaver heads provide natural soft tissue properties (Thiel, 1992) and have been described as a suitable model for middle ear research (Stieger et al., 2012; Guignard et al., 2013).

### Experimental protocol

The experimental protocol is summarized in Table 1. The protocol comprises the successive experimental steps (1a–4b), the corresponding clinical implications, details of the sound source, the measurement method and the corresponding figures. In detail:

**Table 1:** Experimental steps.

**No**.	**Experiment**	**Clinical implication**	**Sound source/measurement**	**Figures**
1a	Mastoid cavity open, TM intact. Systematic variation of *d*, x, y, z. x, y: changed, z = 0, *d* = 2 mm.	Individual variation of the position of microphone d and sound source x, y, z	ER-2/ER-7C	Figures 2, 3A
b	x = 0, *y* = 0, z = 0, 5, 10 mm, *d* = 2 mm.			
c	x = 0, *y* = 0, z = 10 mm, *d* = 7, 12, 17 mm.			
2a	Mastoid cavity closed with skin flap, TM intact. Fixed d & x, y, z.	Post-operative situation	ER-2/ER-7C	Figures 2, 3B
b	Mastoid cavity open, TM elevated. Fixed d & x, y, z.	Intra-operative situation	ER-2/ER-7C	Figures 2, 3B
3a	Mastoid cavity open, TM elevated. Stapes removed, DACS actuator in place.	Intra-operative situation	DACS actuator/ER-7C & LDV	Figure 4
b	Mastoid cavity open, TM elevated. Piston prosthesis inserted.	Intra-operative situation	DACS actuator/ER-7C & LDV	Figure 4
4a	Mastoid cavity open, TM elevated. Misplacement of a working DACS actuator.	Intra-operative situation	DACS actuator/ER-7C & LDV	Figure 5A
b	Mastoid cavity open, TM elevated. Insertion of a defective DACS actuator.	Intra-operative situation	DACS actuator/ER-7C & LDV	Figure 5B

*TM, Tympanic membrane; ER-2, Loudspeaker; ER-7C, Probe Microphone for sound pressure measurement in the external auditory canal P_EAC_, LDV, Laser Doppler vibrometry for velocity measurement of actuators vibration rod tip. D, distance of the probe microphone (ER-7C) to the tympanic membrane; x, y, z, position in three dimensions of the loudspeaker (ER-2)*.

**Figure 2:**
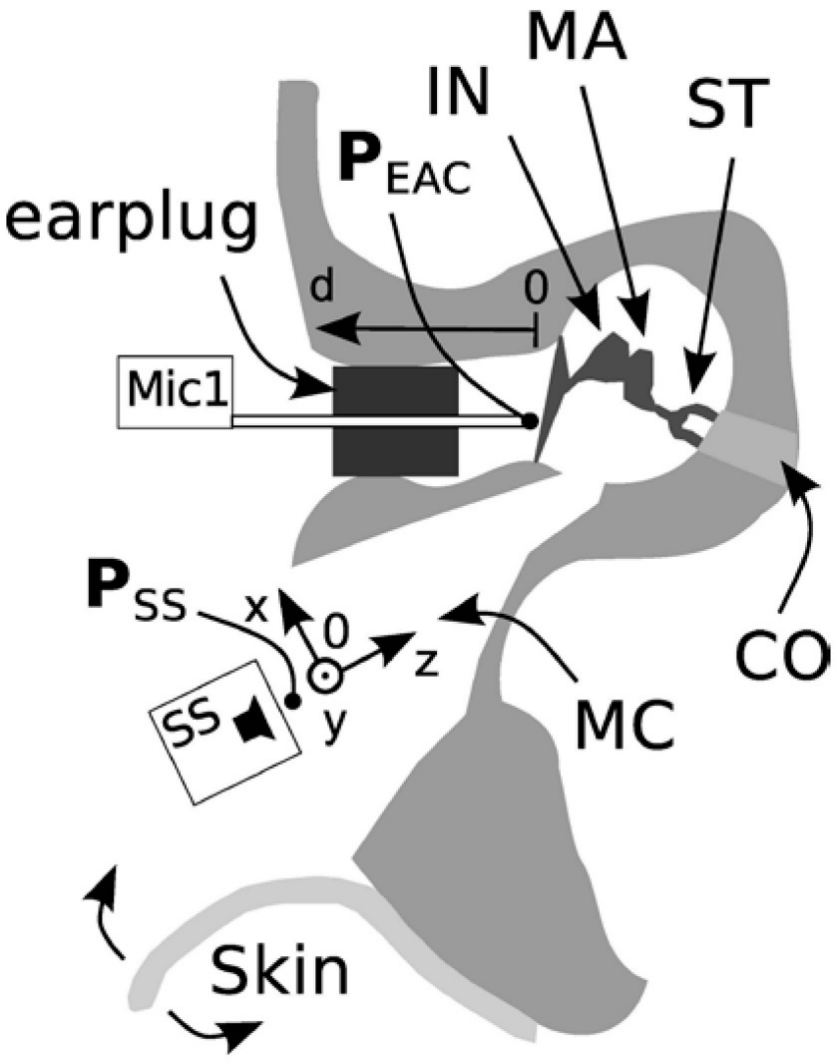
Specimen preparation and measurement setup (axial cut, top is frontal). The pressure in the ear canal, P_EAC_, was measured after a mastoidectomy with an intact ossicular chain (IN, incus; MA, malleus; ST, stapes) and tympanic membrane. The external auditory canal (EAC) was closed using a foam earplug. The loudspeaker ER-2 (SS) was placed initially at position 0 and displaced along the x, y, and z axes. The tip of probe microphone was initially positioned 2 mm from the tympanic membrane and displaced along the d-axis (Mic1). The skin flap was used to close the mastoid cavity (MC). CO, cochlea.

The mastoid bone was drilled out to create a cavity which provided sufficient space for later placement (see Step 3–4) of the DACS actuator (Figure 1). Sound was applied in the mastoid cavity. P_EAC_ was measured with a probe microphone (ER-7, Etymotic Research, Elk Grove Village, Illinois). The ear canal was plugged throughout all experiments with a perforated foam earplug (E-A-RLink 3C, Aearo Technologies Auditory Systems, Indianapolis IN, USA). The tympanic membrane remained intact. We systematically examined the influence of the distance (d) from the tip of the probe microphone to the tympanic membrane and position in three dimensions (x, y, z) of the loudspeaker ER-2 (Etymotic Research Inc., Elk Grove Village, IL, USA) inside the mastoid cavity in five ears. (1a) The loudspeaker was displaced with micromanipulators (Thorlabs Inc., Newton, NJ, USA) in x-y plane (parallel to the mastoid surface) at five positions i.e., mid position of the cavity (x = 0 mm, y = 0 mm) and most anterior (positive x axis direction), posterior (negative x axis direction), superior (positive y axis direction), and inferior positions (negative y axis direction). The extremities in the x and, y direction were up to ±5 mm in all specimens.

(1b) Initially we measured at the lateral surface level (z = 0 mm) of the mastoid cavity (MC) and repeated then measurement at *z* = 5 and 10 mm (Figure 2) again using a micromanipulator. P_EAC_ was measured at *d* = 2 mm from the tympanic membrane using the probe microphone. To measure the initial position of the tip of the probe tube, we determined the distance from the tragus to the tympanic membrane with a surgical probe. This distance −2 mm was transferred onto the ER7-C probe microphone tube from the tip to a marker ring. The ear canal was plugged with a standard foam plug (ER3-14) for insert phones. The ER3-14 has a black tube where typically sound is applied. In our experiment, the black tube was used as a feed through for the microphone probe tube. The black tube was cut off at the level of the tragus and the microphone probe tube was inserted up to the marker ring. The microphone probe tube was thereby guided along the black tube of the ER3-14 such that the tip of the flexible probe tube remained in a free position within the ear canal. Additionally, the ear canal was in an approximately vertical position (corresponding to the patients head position in DACS surgery), which further reduces the risk of contact of the flexible probe tube with the wall of the external auditory canal. (1c) Hereafter the loudspeaker was set in the middle of the mastoid cavity (x, y = 0, *z* = 10 mm) while the distance of the probe microphone from the tympanic membrane was manually increased in 5 mm steps (7, 12, and 17 mm). With the variation of the loudspeaker in the x, y, z positions in the mastoid cavity and the variations of the probe microphone distance d to the tympanic membrane we aimed to cover the clinical relevant variations.

(2a) The influence of an open or closed mastoid cavity with skin was measured with the loudspeaker at the same mid position in the mastoid cavity (x, y = 0 mm, z = 10 mm) and the probe microphone *d* = 2 mm distance from the tympanic membrane as described in Section Introduction. In the first measurement the mastoid cavity was open and the tympanic membrane remained intact. Hereafter we measured after closure of the mastoid cavity with the skin flap. This situation mimics partly the acoustic post-operative situation. In the real post-operative situation the ossicular chain would be interrupted and a piston prosthesis coupled to the actuator after intraoperative elevation and closure of the tympanic membrane. Functional restoration of an elevated tympanic membrane in a cadaver specimen is difficult to achieve. The influence of the skin flap was therefore solely measured with intact tympanic membrane (Figure 2).

(2b) The tympanic membrane was then elevated and the skin from the mastoid cavity removed. This situation mimics the acoustic intraoperative situation.

(3a) After testing the influences of the loudspeaker and probe microphone positions, as well as and open vs. closed cavities, DACS actuator implantation was performed according to the surgical procedure described in Hausler et al. (2008) i.e., the tympanic membrane was elevated, and the mastoid cavity was opened with the stapes completely removed (stapedectomy; Figure 1A). The size of the DACS actuator and the fixation system generally does not permit visually controlling, if the vibrating rod is moving freely without touching surrounding bone (Figure 1A). The DACS actuator was driven with a voltage of 316 mV_rms_. The vibration of artificial incus was measured using LDV through the ear canal with the tympanic membrane elevated without the need of reflective beads. P_EAC_ (*d* = 12 mm) and V_act_ were sequentially measured in two different conditions. In the first condition, the actuator was unloaded, i.e., without prosthesis. This condition corresponded to the status during the surgical procedure directly after insertion and clamping of the actuator with the fixation system. (3b) In the second condition, a piston prosthesis (0.6 mm width, 4.5 mm length, Teflon piston, Richards Medical Co. Memphis, TN, USA) was inserted into the opened oval window and crimped to the artificial incus.

(4a) We tested two potential failures that may occur during implantation surgery in one ear. In the first case, the motion of the rod was limited by contact to the surrounding tissue. Such manipulation can potentially result in a defective actuator and is not always reversible. (4b) In the second failure mode, we implanted a “damaged” actuator with a resonance frequency out of the specification range in a single specimen. Again the V_act_ and P_EAC_ was measured.

### Signal generation, acquisition, and processing

We used a UPV Audio Analyzer (Rohde & Schwarz, Munich, Germany) for signal generation and data acquisition. We stimulated with 50 and 90 pure sinusoidal tones in a logarithmically equidistant scale ranging from 0.1 to 10 kHz at 1.5 V_rms_ for the loudspeaker and 316 mV_rms_ for the DACS actuators respectively. We used two DACS prototype actuators. V_act_ was measured with a calibrated HLV-1000 Laser Doppler Vibrometer (Polytec GmbH, Waldbronn, Germany). The sound-pressure level was measured with an ER-7C probe microphone (Etymotic Research Inc., Elk Grove Village, IL, USA). An ER-2 loudspeaker (Etymotic Research Inc., Elk Grove Village, IL, USA) was used for testing the influence of the position of the sound source, skin flap and tympanic membrane (Table 1, experiments 1, 2). The ER-2 loudspeaker provides higher sound pressures with flat response in a wider frequency range than the sound emission from the actuator. Furthermore, to test the influence of different sound source positions in the mastoid cavity on P_EAC_, the ER-2 can stimulate at positions beyond the clinically possible placement range of an implanted DACS actuator. Data acquisition of P_EAC_ and Vact was performed with a sampling rate of 48 kHz. For each stimulation frequency, a selective root mean square (RMS) value at the stimulation frequency was measured using a bandpass filter. For frequencies below 1,000 Hz the bandwidth of the filter was fixed to 10 Hz, for higher frequencies the bandwidth of the filter was ±1% of the stimulation frequency. The RMS value measurement at the filter output started after expiry of the filter settling time. The measurement was averaged from 3 consecutive measurements at each frequency. The sequence was repeated twice, and typically showed reproducibility within 2 dB along the considered frequency range. The measurement time for measuring of one spectrum of the DACS actuator typically took 60–90 s which was estimated as acceptable for the data acquisition of an intraoperative test. We used the same data acquisition setup for both V_act_ and P_EAC_ measurements. Statistical analyses were conducted, and figures were prepared with Prism 5 (GraphPad Software, Inc., La Jolla, CA, USA).

The background operating room noise was measured in the local ENT operation theater. Sound pressure levels at 0.5, 1, 2, 3, 4, and 5 kHz were measured with a calibrated ½-inch condenser microphone (FF4133, Brüel & Kjær, Nærum, Denmark) at a distance of 10 cm from the patients ear. The active noise sources were the operating room air conditioning system, anesthesia system (Dräger, Primus 35), heating cover (CS2 hyperthermia system) and facial nerve monitoring system (Neurosign 100, Innomed).

## Results

**Figure 3:**
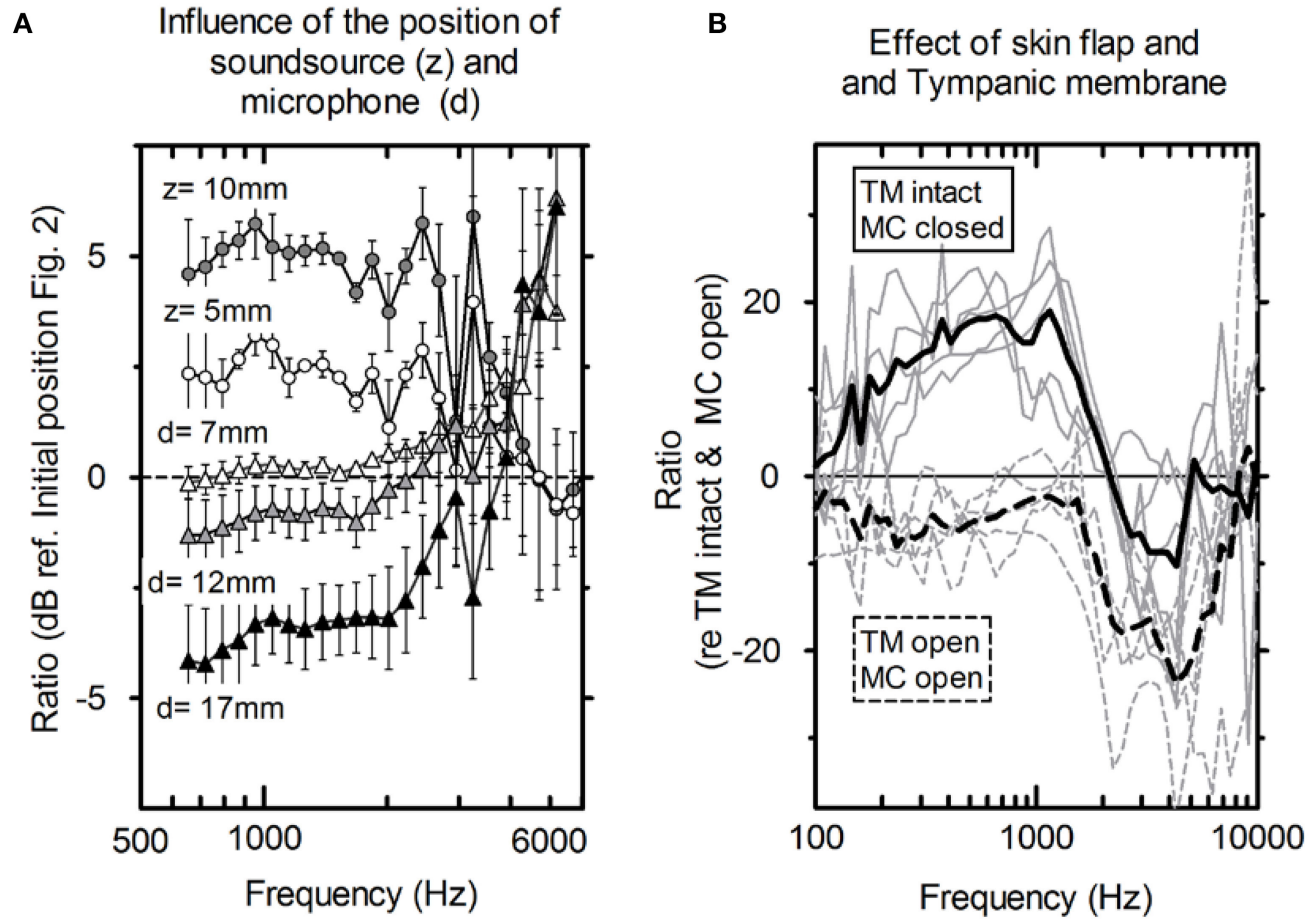
**(A)** Average influence of the distance of the microphone to the tympanic membrane (circles, *n* = 5) and actuator position toward the depth of the mastoid cavity (triangles, *n* = 5) and the standard deviation. **(B)** P_EAC_ variations. Solid lines: mastoid cavity (MC) closed with skin flap and intact tympanic membrane (TM); dashed lines: mastoid cavity (MC) open and tympanic membrane is elevated (TM); gray lines: individual measurements; black lines: averaged (*N* = 5).

The influence of the loudspeaker position (Figure 2) toward the depth of the cavity (z) and the distance of the microphone to the tympanic membrane (d) is shown in Figure 3A. For axial displacement of the actuator along the z-axis toward the middle ear cavity, a small difference in P_EAC_ of ~2.5 dB at z = 5 mm was observed. At z = 10 mm, the difference was 5 dB on average. Moving the loudspeaker in the x-y plane (along the mastoid surface) minimally affected P_EAC_ (range of −0.5 to 2.5 dB) and is therefore not shown. The average P_EAC_ at *d* = 7 mm differed less than one standard deviation (*n* = 5) from the initial position (*d* = 2 mm in front of the tympanic membrane). At 12 mm, the difference was below 2.5 dB.

Figure 3B shows the average difference and standard deviation between the intra-operative and post-operative P_EAC_ in five specimens (see Table 1: 2a, b). The two main differences are the closure of the mastoid cavity with the skin flap and the elevation of tympanic membrane closure. Closing the mastoid cavity with the skin flap induced an increase in P_EAC_ for frequencies below 2 kHz with a maximum of 20 dB at 600 Hz. Above 2 kHz, an attenuation of 10 dB was observed. P_EAC_ for a closed tympanic membrane is generally lower that for an elevated tympanic membrane. It is ~5 dB below 1.3 kHz and by 20 dB for frequencies 2–5 kHz. On average, the combined effect of both was an increase in magnitude from 0 dB at 0.1 kHz up to ~10 dB, near 1.2 kHz, further dropped to 0 dB, near 1.6 kHz, and showed a maximum attenuation of −20 to −30 dB between 2 and 7 kHz.

**Figure 4:**
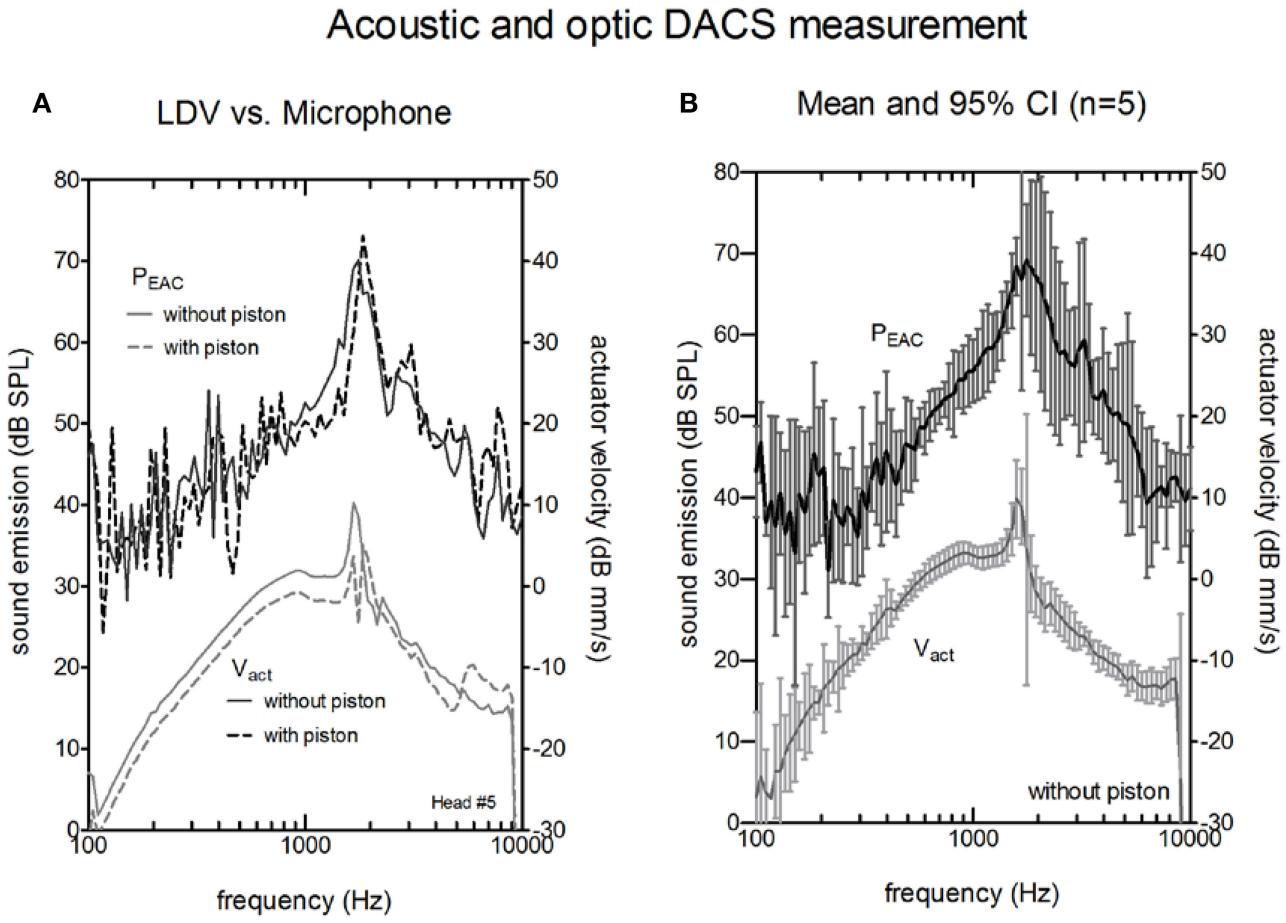
Actuator velocity of the vibrating rod V_act_ (lower curves, right y-axis) and the resulting sound pressure in the ear canal P_EAC_ (upper curves, left y-axis). **(A)** A single measurement without piston (solid lines) and with piston (dashed lines) actuators using a Laser Doppler Vibrometer (LDV). **(B)** Average magnitude of P_EAC_ and V_act_ of the five actuators without a piston. The bars indicate the confidence interval (CI).

**Figure 5:**
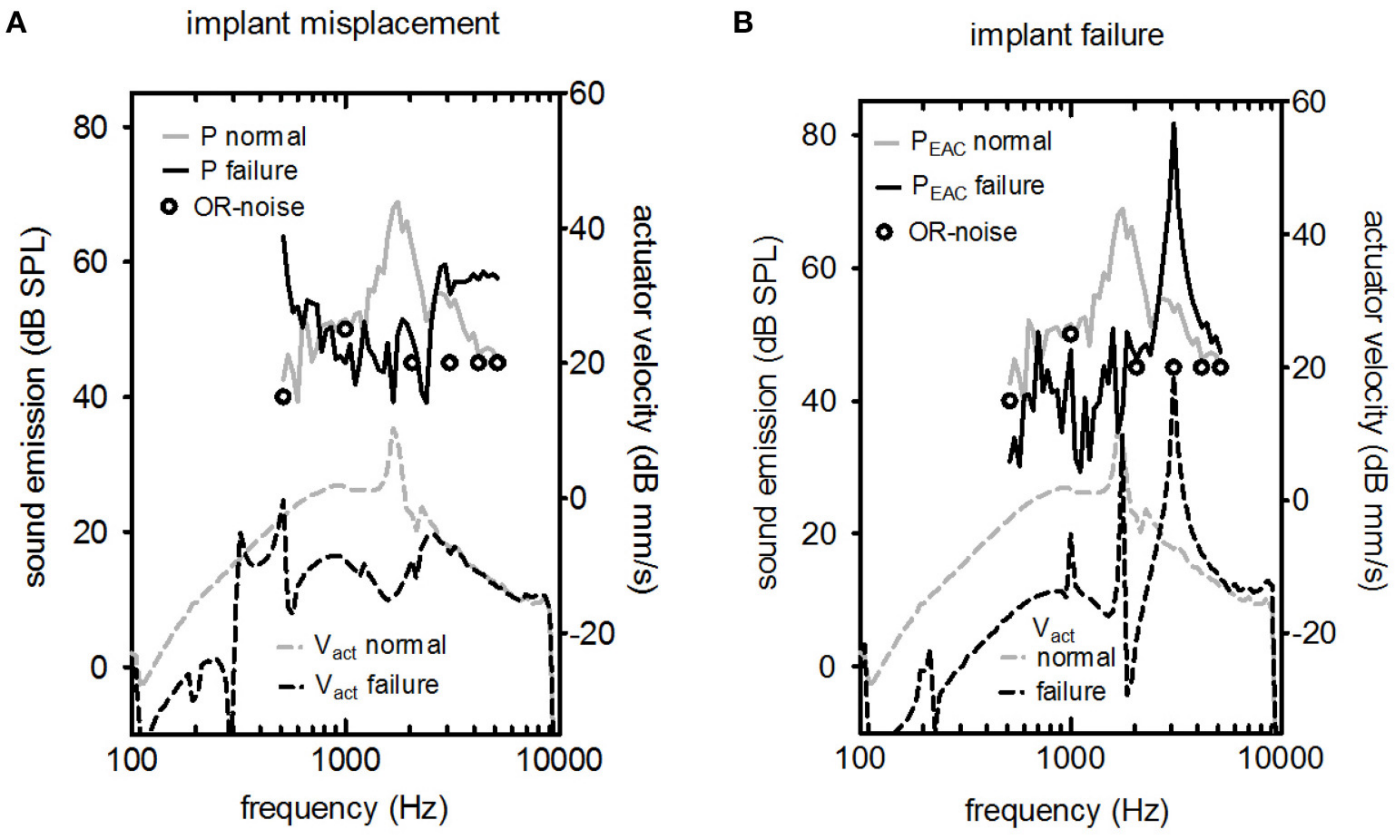
LDV (Laser Doppler velocity) measurement of V_act_ (upper part) and the resulting sound pressure in the ear canal P_EAC_ (lower part) for two cases of failure (excitation level 316 mV). **(A)** The actuator's vibrating rod was in contact with the surrounding bone, and **(B)** the actuator had a non-standard response. In both graphs, the typical background noise in an operating room (OR) during hearing implant surgery is shown (circle symbols).

The comparisons between the acoustical (probe microphone in the ear canal) and the optical (LDV at the actuator the artificial incus) methods for the intra-operative test are shown in Figures 4, 5. Figure 4A shows the sound emission of the actuator and the corresponding V_act_ with and without attached piston prosthesis in a single example. V_act_ and P_EAC_ rose from 100 Hz to the resonance frequency of the actuator (~1,500 Hz) and decreased thereafter. The resonance frequency was clearly detectable in both measurements. With attached piston prosthesis, V_act_ and P_EAC_ were reduced. Compared to P_EAC_ measurement, the LDV data showed a smoother response.

Figure 4B shows the averaged V_act_ and P_EAC_ for five cadaver heads. LDV measurements provided stable and reproducible results within the entire frequency range (100–10,000 Hz). Sound measurements with an SNR higher than 6 dB were typically observed between 800 Hz and 8,000 Hz.

In the microphone measurements, the resonance of the actuator was observed at the same frequency as the LDV measurements. The magnitude at resonance ranged between 66.6 and 77.45 dB SPL, and the peak was generally broader than the peak for LDV measurements. P_EAC_ correlated significantly with V_act_ (Pearson correlation, *p* < 0.05) for frequencies between 800 and 8000 Hz in all measurements. The confidence interval for the acoustic measurements (median 5 dB) was larger than for the optical measurements (median 1.5 dB).

The effects of misplacement and actuator failure are shown in Figure 5. Implant misplacement, i.e., contact of the vibrating element with the surrounding bone, changed the characteristic shape drastically for acoustic and optic measurements for implant misplacement (Figure 5A). The peak at the resonance frequency was suppressed. The level of the acoustic signal was close to the background noise level in the operating room. Figure 5B shows a normal actuator compared to a malfunctioning actuator with a frequency response outside the manufacturer's specifications. Elevated resonance frequency was detected for V_act_ and P_EAC_.

## Discussion

The actuator of the DACS implantable hearing device emits sound across the mastoid cavity to the middle ear cavity and the EAC. We measured these emissions in anatomical whole head cadaver specimens in experimental intra- and post-operative scenarios to evaluate the performance of the actuator.

### Intra-operative P_EAC_

P_EAC_ generated by the DACS implant was higher than the typical background noise measured during an intra-operative test (Figure 5) for the frequency range of 800–8,000 Hz. The resonance frequency was identified in a typical single measurement as well as by the mean of multiple measurements (Figure 4). A change in the position of the loudspeaker and the microphone by 10 mm within the EAC resulted in a linear shift in P_EAC_ of <5 dB between 1 and 3 kHz (Figure 3A).

Coupling the actuator to a piston prosthesis added mass and modified V_act_. As a result, the magnitude at the resonance frequency was reduced (Figure 4A). As reported (Chatzimichalis et al., 2012), the coupling quality, especially for manually crimped pistons, can induce velocity differences of up to 3.5 dB.

Two important objectives of the intra-operative test are the detection of implant failure or misplacement. There are two relevant time points to perform an intra-operative integrity test. First, the test was conducted directly after insertion and fixation of the actuator into its final position. In this situation, surgeons could check for abnormal actuator resonance frequency so that they could perform fine adjustments in case of misplacement or exchange the actuator if implant failure occurs (Figure 5). Second, a test was conducted after coupling the stapes prostheses. In this case, a positive result would show a working device after all manipulations of the actuator, including crimping of the piston prosthesis on the artificial incus. However, a negative test result would require additional manipulations with an increased risk of intra-cochlear damage because the piston prosthesis was already placed.

One reason to measure the integrity before coupling the piston prosthesis is that no mechanical stimulation of the inner ear is typically applied. In contrast, when the prosthesis is coupled, the actuator mechanically stimulates the cochlear fluid. We used 316 mV for our experiments because this value resulted in a measurable sound emission of the DACS, which was higher than operating room noise for the frequencies around resonance. In a previous study, Chatzimichalis (Chatzimichalis et al., 2012) found that 300 mV at the DACS actuator provided more than 110 dB ESPL to the inner ear. In clinical studies (Zwartenkot et al., 2014), the DACS was shown to be the most powerful acoustic stimulator. Therefore, it may be advisable to perform intra-operative measurements before the attachment of the stapes prostheses to the artificial incus to avoid intra-cochlear damage (Chatzimichalis et al., 2012).

We used disposable foam earplugs in this experimental study. In the clinical scenario, when measurements are made with a microphone in the ear canal while the tympanic membrane is elevated, sterility concerns emerge. The microphone cable and pre-amplifier could be packed in a sterile plastic cable sheath, such as the one used for intra-operative measurement of cochlear implants. The disposable earplug and microphone probe tube should be sterilized prior to the procedure.

In the intra-operative scenario in which the tympanic membrane and skin flap were open and the actuator was uncoupled, a resonance peak close to 1.3 kHz indicated that the implanted actuator functioned properly (Figure 5B). LDV measurement of V_act_ allowed for quantitative estimation of the equivalent hearing level (Chatzimichalis et al., 2012). We showed that we could also assess the transfer function of an implanted DACS actuator with a microphone. However, the confidence interval for the acoustic measurement was larger than for LDV measurements and depended not only on the actuator but also on the setup and individual anatomy. Therefore, we could not estimate the equivalent hearing level based on acoustic measurements only.

### Post-operative P_EAC_

Post-operative P_EAC_ measurements of the DACS emission could potentially be used to monitor the function of the implant non-invasively. While coupling to the piston prosthesis had a marginal effect, elevation of the tympanic membrane, and closure of the mastoid cavity had a drastic impact on the microphone measurements. The mastoid cavity mostly influenced frequencies below 2,000 Hz, while elevation of the tympanic membrane mainly influences frequencies above 1,000 Hz (Figure 3B). Therefore, measurements of P_EAC_ that are performed intra-operatively may significantly differ from measurements performed post-operatively. However, the influence of elevation of the tympanic membrane was small at lower frequencies and near the actuator resonance, whereas closing the mastoid cavity increased the output to the ear canal at the same frequency. As a result, we expected P_EAC_ to be well above the noise level at frequencies between 0.1 and 2 kHz in the post-operative scenario. In addition, post-operative measurements could be performed in an audiometry test booth in which the background noise is lower than in the operating room, allowing for stimulation levels below 110 dB ESPL.

Our postoperative results are based on measurements with an intact tympanic membrane and ossicular chain because the postoperative healing of the tympanic membrane after the stapedectomy is simulated only with difficulty in human Thiel-embalmed specimens. Since the “healed tympanic membrane” with the surgically-reconstructed middle ear is different from the intact tympanic membrane with the intact middle ear, the results in this study may differ from the real postoperative results. This is especially the case considering our protocol of reverse stimulation with the sound source in the mastoid cavity.

However, considering a fact that in case of forward stimulation, the ear-canal pressure even with large perforations is fully recovered only by paper-patch on the perforation (O'Connor et al., 2008; Röösli et al., 2012), the difference may be presumed to be small.

## Conclusion

The actuator of the DACS implantable hearing device acted as a sound source in the mastoid cavity. The emitted sound could be measured in the ear canal and used for an intra-operative function test of the actuator. With closed the skin over the mastoid cavity and closed tympanic membrane, the shape of the frequency response of P_EAC_ was systematical different than in the intra-operative scenario. Acoustical measurement is an alternative to optical (LDV) measurement for the intra-operative and post-operative evaluation of the DACS functionality.

## Author contributions

CS: PI, experiments, data analysis, manuscript preparation; YA and CC: Experiments; MC: Manuscript review, Co-PI; AA: Co-PI, experiments, data analysis, manuscript preparation.

### Conflict of interest statement

The authors declare that the research was conducted in the absence of any commercial or financial relationships that could be construed as a potential conflict of interest.
